# The prevalence of dental caries and associated factors among secondary school children in rural highland Vietnam

**DOI:** 10.1186/s12903-021-01704-y

**Published:** 2021-07-16

**Authors:** Nguyen Van Chuyen, Vu Van Du, Nguyen Van Ba, Dao Duc Long, Ho Anh Son

**Affiliations:** 1 Military Hygiene Department, Vietnam Military Medical University, No.160 Phung Hung Street, Ha Dong, Hanoi, Vietnam; 2 Quality Management Department, Requested Service Department, National Hospital of Obstetrics and Gynaecology, No. 43 Trang Thi Street, Hoan Kiem, Hanoi, Vietnam; 3 The 103 Military Hospital, Vietnam Military Medical University, No.160 Phung Hung Street, Ha Dong, Hanoi, Vietnam; 4 Politic Department, Vietnam Military Medical University, No.160 Phung Hung Street, Ha Dong, Hanoi, Vietnam; 5 Institute of Biomedicine and Pharmacy (IBP), Vietnam Military Medical University, No.160 Phung Hung Street, Ha Dong, Hanoi, Vietnam

**Keywords:** Dental caries, Prevalence, Secondary school children, Rural, Highland, Vietnam

## Abstract

**Background:**

To determine the prevalence of dental caries in primary and permanent teeth and identify factors associated with dental caries among secondary school children in rural highland Vietnam.

**Methods:**

This was a cross-sectional study that included 1985 secondary schoolchildren. Dental examination was performed at school using World Health Organization criteria. Data collection on demographic characteristics and knowledge, attitude, and practices related to dental caries was conducted by interviewing children. Descriptive and inferential statistics using a multivariate logistic regression model were applied.

**Results:**

Prevalence of caries in primary and permanent teeth was 41.1 and 68.9 %, respectively. Prevalence of caries in primary teeth in the age group 11–12 years old (59.4 %) was significantly higher than in children in the age group of 13–14 years (27.8 %; *p* < 0.01). Factors associated with dental caries in primary teeth were age group of 11–12 years, belonging to the Jarai ethnic group, and having inadequate knowledge or attitude related to dental caries. Factors associated with dental caries in permanent teeth were having insufficient knowledge, attitude, and practices related to dental caries.

**Conclusions:**

The prevalence of dental caries in primary and permanent teeth was high among secondary school children in Vietnam’s rural highlands. It is recommended that interventions focus on younger secondary school children and the Jarai minority ethnic group, and that interventions should emphasize improving knowledge, attitudes, and practices related to dental caries.

## Background

Dental caries, also known as tooth decay, is caused by damage to the tooth enamel [1]. Dental caries is a widely observed chronic disease in humans, and anyone can be at risk of dental caries throughout their lifetime [1, 2]. Importantly, dental caries is the most common disease associated with oral health in school-aged children [3–7]. According to the World Health Organization (WHO), dental caries affects 60–90 % of schoolchildren globally, mainly in developing countries [8–10]. While dental caries tends to be well-controlled in developed countries, its prevalence is increasing in low- and middle-income countries [9]. Children with dental caries suffer pain that may lead to difficulties eating, sleeping, and communicating, and its presence may affect concentration in school, thus influencing educational development. If not detected and promptly treated, dental caries leads to severe pain and infection [11], and, if left untreated, severe dental caries may require costly surgical intervention [12, 13].

Vietnam has made remarkable progress in improving public health over the past few decades. However, an effective oral care strategy is still undeveloped and existing strategies have not met the needs of the population [14]. Although previous studies in Vietnam indicated a high prevalence of dental caries in primary and permanent teeth in children [14–17], these studies used outdated datasets or small sample sizes, or focused only on urban areas. To date, no studies regarding the prevalence of dental caries in children in rural highland areas of Vietnam have been conducted. Similarly, few studies have been conducted that investigated the factors associated with dental caries that include patient knowledge, attitudes, and practices related to dental caries. Thus, this study aims to determine the prevalence of dental caries in primary and permanent teeth and identify factors associated with dental caries in secondary school children in rural highland Vietnam.

## Methods

### Study design and setting

This was a cross-sectional study conducted from August through October 2017 in Gia Lai province, Vietnam. Gia Lai is located in the central highland region and is the second-largest province of Vietnam by area, and the majority of its population lives in rural and mountainous areas. In 2019, Gia Lai had a population of approximately 1.5 million people. In Vietnam, the Kinh is a majority ethnic group, whereas the Jarai is a minority ethnic group. This study was conducted in two rural districts in Gia Lai that are mainly inhabited by the Kinh and Jarai ethnic groups. The two rural districts were randomly selected among 14 rural districts of Gia Lai province. Study participants included secondary school students in the two districts. Approval to conduct the study was obtained from the Institutional Review Board of the Vietnam Military Medical University (decision no. 350/2016/YTCC-HD3, dated 29/12/2016). All procedures performed in the present study involving human participants were in accordance with the ethical standards of the institutional and/or national research committee and with the declaration of Helsinki 1975, which was revised in 2000.

### Sample size and sampling

Inclusion criteria were children studying at secondary schools who obtained permission from their parents or guardians to participate in the study. We excluded children who had any chronic debilitating disease or who had a fever on the survey day. We calculated the sample size according to the formula for estimating a population proportion with specified absolute precision suggested by the WHO [18], and included an anticipated proportion of dental caries among children of 70 % based on a pilot study of 50 children in a single secondary school. The school surveyed in the pilot study was in the study area but was not included in this study. We estimated that the minimum sample size needed for this study was 323 children based on a significance level of 0.05 and an absolute precision of 0.1. Since secondary schools in Vietnam include four grades (grades six through nine), we multiplied our sample size by four to generate a total target sample size of 1292 children. Allowing for a non-response rate of 30 %, we estimated that we would need at least 1,845 children for this study. We invited 2057 children from six randomly-selected secondary schools from 24 secondary schools in two rural highland districts of Vietnam. The participation rate was 96.5 % (n = 1985), with a small number of children not participating due to non-consent by the child or their parents or non-attendance at school (n = 72).

### Variables

We considered the presence of dental caries in primary or permanent teeth to be an outcome variable in this study. To identify caries, we performed a dental examination of children at the selected schools using the WHO dental caries diagnosis guidelines to identify the number of decayed teeth, teeth missing due to caries, and filled teeth, for both primary teeth and permanent teeth (dmft/DMFT) [19]. We then calculated dmft and DMFT indices for primary and permanent teeth, respectively, to describe the status of children’s dental caries, with dental caries in primary and permanent teeth defined as dmft > 0 and DMFT > 0, respectively.

We collected independent variable data including sex (girl or boy), age group (11–12 years old or 13–14 years old), ethnicity (Kinh or Jarai), and father’s and mother’s occupations (official or worker, farmer, or freelancer). We also collected data regarding study participants’ knowledge, attitude, and practices related to dental caries through interviews. Interviews consisted of ten questions for each group of topics about knowledge, attitude, and practices related to dental caries for a total of 30 questions. Correct answers garnered one point, and incorrect answers garnered zero points. Scores (out of 10) for knowledge, attitudes and behaviors were summed for each child. Knowledge, attitudes and behaviors were rated as “good” if they scored 8 or more. Independent variables for multivariate analysis were good knowledge (yes or no), good attitude (yes or no), and good practices (yes or no).

### Statistical analysis

We present quantitative variables as means and standard deviations and qualitative variables as frequencies and percentages. Differences between groups were analyzed according to Chi-square tests (qualitative variables). We used multivariate logistic regression analysis to calculate adjusted odds ratios (AOR) and identify any factors associated with dental caries. We conducted all statistical analyses using STATA version 14.0 (Stata Corp, College Station, TX, USA), and differences were considered significant at p-values < 0.05.

## Results

**Fig. 1:**
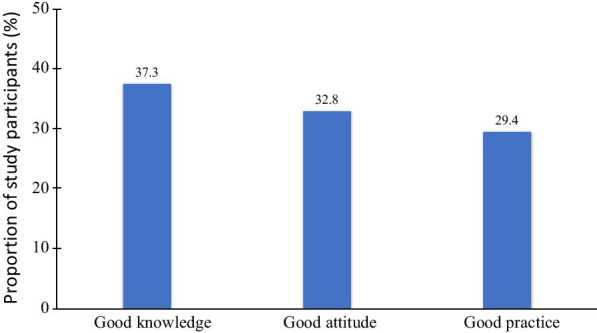
Proportion of secondary school children with good knowledge, attitude, and practice related to dental caries

A total of 1,985 secondary school children aged 11 through 14 years (in grades six through nine) were enrolled in the study. Table 1 indicates the characteristics of participants in this study. The proportions of girls to boys and that of children aged 11–12 years or 13–14 years were slightly different. Similarly, more children from the Jarai minority ethnic group were included than were children from the Kinh majority ethnic group. Figure 1 illustrates the proportion of children with good knowledge, good attitude, and good practices related to dental caries.

**Table 1:** Characteristics of study participants (n = 1,985)

	N (%)
*Sex*	
Girl	1074 (54.1)
Boy	911 (45.9)
*Age group*	
11–12 years	1153 (58.1)
13–14 years	832 (41.9)
*Ethnicity*	
Kinh	874 (44.0)
Jarai	1111 (56.0)
*Father’s occupation*	
Official or worker	721 (36.3)
Farmer	609 (30.7)
Freelancer	655 (33.0)
*Mother’s occupation*	
Official or worker	711 (35.8)
Farmer	598 (30.1)
Freelancer	676 (34.1)

Table 2 shows the prevalence of dental caries among children according to various factors. The overall prevalence of caries in primary and permanent teeth was 41.1 and 68.9 %, respectively. There were no significant differences in the prevalence of caries in primary or permanent teeth between boys and girls or prevalence in permanent teeth between the age group of 11–12 years and 13–14 years (all p > 0.05). However, the prevalence in primary teeth in the age group of 11–12 years (59.4 %) was significantly higher than in children in the age group of 13–14 years (27.8 %; p < 0.01). There were significant differences in the prevalence of caries in primary and permanent teeth between children who did and did not have good knowledge, attitude, and practices related to dental caries (all p < 0.01).

**Table 2:** Prevalence of dental caries among secondary school children according to various factors

Factors	Dental caries			
	Primary teeth		Permanent teeth	
	N (%)	*p* value	N (%)	*p* value
Total	815 (41.1)	NA	1,368 (68.9)	NA
*Sex*				
Girl	440 (41.0)	0.93	749 (69.7)	0.39
Boy	375 (41.2)		619 (68.0)	
*Age group*				
11–12 years	494 (59.4)	< 0.01	576 (69.2)	0.80
13–14 years	321 (27.8)		792 (68.7)	
*Ethnicity*				
Kinh	339 (38.8)	0.68	598 (68.4)	0.67
Jarai	476 (42.8)		770 (69.3)	
*Good knowledge*				
Yes	112 (15.2)	< 0.01	201 (27.2)	< 0.01
No	703 (56.4)		1,167 (93.7)	
*Good attitude*				
Yes	72 (11.1)	< 0.01	114 (17.5)	< 0.01
No	743 (55.7)		1,254 (93.9)	
*Good practice*				
Yes	74 (12.7)	< 0.01	109 (18.7)	< 0.01
No	741 (52.9)		1,259 (89.8)	
*Father’s occupation*				
Official or worker	286 (39.7)	0.64	493 (68.4)	0.35
Farmer	255 (41.9)		433 (71.1)	
Freelancer	274 (41.8)		442 (67.5)	
*Mother’s occupation*				
Official or worker	320 (45.0)	< 0.01	535 (75.3)	< 0.01
Farmer	211 (35.3)		368 (61.5)	
Freelancer	284 (42.0)		465 (68.8)	

NA: Not applicable

Table 3 indicates results of the multivariate logistic regression analysis. Factors that were determined to be significantly associated with dental caries in primary teeth were age group of 11–12 years (AOR = 6.2; 95 % confidence interval [CI] = 4.9–7.9), belonging to the Jarai ethnic group (AOR = 1.4; 95 % CI = 1.2–1.8), and children with insufficient knowledge (AOR = 3.4; 95 % CI = 2.5–4.6) and attitude (AOR = 5.6; 95 % CI = 3.2–9.7) related to dental caries. Factors that were significantly associated with dental caries in permanent teeth were children with insufficient knowledge (AOR = 13.5; 95 % CI = 9.5–19.2), attitude (AOR = 14.1; 95 % CI = 8.3–23.9), and practices (AOR = 2.6; 95 %CI = 1.5–4.4) related to dental caries.

**Table 3:** Multivariate logistic regression analysis of dental caries and its associated factors

Factors	Dental caries			
	Primary teeth		Permanent teeth	
	AOR (95% CI)	*p* value	AOR (95 % CI)	*p* value
*Sex*				
Girl	1		1	
Boy	1.1 (0.8–1.3)	0.66	0.9 (0.7–1.3)	0.78
*Age group*				
11–12 years	6.2 (4.9–7.9)	< 0.01	1.1 (0.8–1.6)	0.59
13–14 years	1		1	
*Ethnicity*				
Kinh	1		1	
Jarai	1.4 (1.2–1.8)	< 0.01	1.1 (0.8–1.6)	0.43
*Good knowledge*				
Yes	1		1	
No	3.4 (2.5–4.6)	< 0.01	13.5 (9.5–19.2)	< 0.01
*Good attitude*				
Yes	1		1	
No	5.6 (3.2–9.7)	< 0.01	14.1 (8.3–23.9)	< 0.01
*Good practice*				
Yes	1		1	
No	1.3 (0.8–2.3)	0.32	2.6 (1.5–4.4)	< 0.01
*Father’s occupation*				
Official or worker	1		1	
Farmer	0.8 (0.6–1.03)	0.08	0.7 (0.4–1.03)	0.07
Freelancer	0.9 (0.7–1.3)	0.81	0.7 (0.5–1.1)	0.15
*Mother’s occupation*				
Official or worker	1		1	
Farmer	0.9 (0.8–1.3)	0.95	0.9 (0.6–1.4)	0.70
Freelancer	1.1 (0.8–1.4)	0.60	0.9 (0.6–1.3)	0.48

AOR: Adjusted Odds Ratio

## Discussion

This is the first study to report the prevalence of dental caries and associated factors in Vietnam’s rural highlands, which face many difficulties compared to other regions in Vietnam. We found that the prevalence of dental caries was relatively high, especially in permanent teeth. We also found a difference in the prevalence of dental caries in primary teeth between children of the Jarai and Kinh ethnic groups. Furthermore, children with insufficient knowledge, attitudes, and practices related to dental caries were more likely to have dental caries than those who had good knowledge, attitudes, and practice. This study provides evidence related to dental caries so that policymakers can develop appropriate intervention plans for rural highland areas in Vietnam.

Dental caries is a concerning issue because it is common among children and negatively impacts a child’s quality of life. Caries is a global public health challenge and is continuously studied and documented in various countries. In 2020, the global prevalence of dental caries in primary and permanent teeth was estimated at 46.2 and 53.8 %, respectively, which was considered to be high [20]. Since our study only included children aged 11–14 years, we report a prevalence of dental caries that varies slightly from those results. We do report findings that are consistent with other previous studies, which also indicated a high prevalence of dental caries among children [21–25]. The prevalence of dental caries in our study was higher than that reported by certain previous studies [21, 22, 24, 26], but lower than that reported by other studies in the same age group [23, 25]. This variation in reported prevalence of dental caries could be due to differences in economic conditions or strategies of preventive medicine in response to oral health problems in each country. In addition, differences may be due to differing sample sizes, sample selection methods, and study dates. However, the prevalence of dental caries among children was generally high in previous studies. The prevalence of dental caries tends to be lower in developed countries and higher in developing countries [20, 27], suggesting that concrete and creative strategies still need to be developed to deal with dental caries, especially in developing countries. Furthermore, as dental caries may disproportionally affect disadvantaged groups [28, 29], it is necessary to develop appropriate oral care public health policies that target marginalized socioeconomic groups.

The relatively high prevalence of dental caries that we found is consistent with previous studies conducted in Vietnam [14, 15, 17], although it is slightly lower than the prevalence reported by Do et al., who began collecting data in 1999 [14]. However, when comparing by age group, our findings were quite similar to theirs. This is a considerable issue and suggests that, although there have been many positive socioeconomic changes in Vietnam in the 20 years between that study and this one, existing policies may not have been sufficient to improve public dental health in rural highland areas. Similarly, we found a lower prevalence of dental caries than did two other reports, although those studies focused on children in kindergarten and primary school age groups, who are at increased risk for dental caries [16, 17]. We suggest that, given the number of studies focusing on younger children, future studies should also focus on children of secondary school age.

We also found a significant relationship between dental caries and knowledge, attitudes, and practices related to dental caries. Our results are similar to previous studies that also reported that children who recognized a dental health problem were less likely to develop dental caries [30, 31]. However, like previous studies, we also found that the prevalence of good knowledge, attitude, and practices related to dental caries was low among schoolchildren [17, 32, 33]. In Vietnam, the School Oral Health Promotion Program has been implemented to improve students’ understanding of oral health since 1980. However, Thuy et al. reported concern that the program has not improved oral health behavior among schoolchildren [17], indicating that policymakers should provide more effective plans to enhance oral health behavior among schoolchildren. In addition, water fluoridation rates in communities and schools have remained low, something that, if improved, could reduce the prevalence of dental caries among schoolchildren. Moreover, a relationship between parental behavior and dental caries among schoolchildren has been reported [34], suggesting that there is also a need for oral health education programs that target parents.

Whereas some previous studies revealed differences in the prevalence of dental caries between boys and girls [14–16], we found no differences, as did a study in India [7]. In addition, we observed that the prevalence of dental caries in the Jarai minority ethnic group was slightly higher than that in the Kinh majority ethnic group, although this difference was not statistically significant. In Vietnam, minority ethnic groups are considered to be vulnerable groups, so they often enjoy advantageous social benefits and government health policies, something that may have contributed to a narrowing of the disparity in disease prevalence between the Jarai and the Kinh ethnic groups. Even so, there may be differences in oral disease patterns among minority ethnic groups due to differing economic conditions within households from various groups. Therefore, we suggest that further research is needed in order to better understand inequalities in dental caries among schoolchildren so that more relevant interventions and policies are created in the future [35, 36].

In this study, we found no difference in the prevalence of dental caries in permanent teeth according to age group. This finding differs from previous studies that reported a higher prevalence of dental caries in permanent teeth in older age groups [37, 38]. This difference may be due to the age groups in our study were older than the age groups in previous studies. However, we did observe that the prevalence of dental caries in primary teeth differed according to age group, indicating that younger children had a higher risk of dental caries than did older children. This was also reported in previous studies [14, 37, 38], along with the finding that younger schoolchildren were more likely to have insufficient oral hygiene practices and improper dietary habits, and thus they were more likely to have dental caries.

We also report that the prevalence of dental caries differed according to mothers’ occupations, although we found no difference when analyzing using a logistic regression model, likely due to the fact that most children’s parents were farmers or freelancers and the study having been conducted in rural areas. There is no specific guideline for classifying occupations in Vietnam, so that task was difficult to apply in practice. In addition, because people in rural areas often have many jobs that are usually short-term, parents with very diverse jobs might all have been classified as freelancers. With such occupation demographics, it is possible that parental employment influences children’s dental health [34, 39]. Similarly, it is possible that children from families whose parents work in more stable jobs (e.g., officials or workers) are more likely to receive adequate oral health care. We suggest that more studies are needed in the future to learn more specifically the role of parental occupation in children’s dental health.

We also wish to address some of the limitations of this study. The diagnosis of dental caries based solely on dental examination without using radiography may not detect all cases of dental caries. Although experienced researchers designed the questionnaires about knowledge, attitude, and practices related to dental caries, some children might not have understood the questions, thus affecting our results. Beyond that, the chosen cutoff threshold for determining good and insufficient knowledge, attitudes, and practices may have influenced our results. Furthermore, while parental education may also relate to children’s oral health behavior, given that we interviewed the study participants at school, we did not collect information regarding parental education. It could also be a limitation related to the age examination because some teeth might not fully erupt. Finally, this study is a cross-sectional study and our results should not be interpreted to indicate causality.

## Conclusions

The prevalence of dental caries in primary and permanent teeth was high among secondary school children in Vietnam’s rural highlands, and the majority of surveyed secondary school children suffered dental caries in permanent teeth. Dental caries in primary teeth was associated with belonging to the age group 11–12 years old, belonging to the Jarai ethnic minority group, and having insufficient knowledge and practices related to dental caries. Meanwhile, dental caries in permanent teeth was associated with having insufficient knowledge, attitude, and practices related to dental caries. Therefore, in addition to implementing interventions that focus on younger secondary school children and the Jarai minority ethnic group, future interventions aimed at improving knowledge, attitudes, and practices for secondary school children are essential.

## Data Availability

The datasets used and/or analysed during the current study are available from the corresponding author on reasonable request.

## Abbreviations

AOR: Adjusted odds ratio
dmft/DMFT: Decayed, missing, and filled (primary/permanent) teeth scores
CI: Confidence interval
WHO: World Health Organization

## References

[1] Selwitz RH, Ismail AI, Pitts NB (2007). Dental caries. Lancet.

[2] Pitts NB, Zero DT, Marsh PD, Ekstrand K, Weintraub JA, Ramos-Gomez F (2017). Dental caries. Nature reviews Disease primers.

[3] Veiga N, Pereira C, Amaral O (2015). Prevalence and determinants of dental caries in Portuguese children. Procedia Soc Behav Sci.

[4] Escoffié-Ramirez M, Ávila-Burgos L, Baena-Santillan ES, Aguilar-Ayala F, Lara-Carrillo E, Minaya-Sánchez M, et al. Factors associated with dental pain in Mexican schoolchildren aged 6 to 12 years. BioMed Res Int. 2017. 10.1155/2017/7431301 PMC5480020 28685149

[5] Zhang S, Chau AM, Lo EC, Chu C-H (2014). Dental caries and erosion status of 12-year-old Hong Kong children. BMC Public Health.

[6] Nomura Y, Maung K, Kay Khine EM, Sint KM, Lin MP, Win Myint MK, et al. Prevalence of dental caries in 5-and 6-year-old Myanmar children. International journal of dentistry. 2019;2019. 10.1155/2019/5948379 PMC6512070 31182962

[7] Kumar S, Kumar A, Badiyani B, Kumar A, Basak D, Ismail MB. Oral health impact, dental caries experience, and associated factors in 12–15-year-old school children in India. International journal of adolescent medicine and health. 2017;29(2). 10.1515/ijamh-2015-0041 26360493

[8] Petersen PE, Bourgeois D, Ogawa H, Estupinan-Day S, Ndiaye C (2005). The global burden of oral diseases and risks to oral health. Bull World Health Organ.

[9] Frencken JE, Sharma P, Stenhouse L, Green D, Laverty D, Dietrich T (2017). Global epidemiology of dental caries and severe periodontitis–a comprehensive review. J Clin Periodontol.

[10] Bagramian RA, Garcia-Godoy F, Volpe AR (2009). The global increase in dental caries. A pending public health crisis. Am J dent.

[11] Ferraz NKL, Nogueira LC, Pinheiro MLP, Marques LS, Ramos-Jorge ML, Ramos-Jorge J (2014). Clinical consequences of untreated dental caries and toothache in preschool children. Pediatr Dent.

[12] Listl S, Galloway J, Mossey P, Marcenes W (2015). Global economic impact of dental diseases. J Dent Res.

[13] Petersen PE. The World Oral Health Report 2003: continuous improvement of oral health in the 21st century–the approach of the WHO Global Oral Health Programme. Community Dentistry and oral epidemiology. 2003;31:3–24. 10.1046/j..2003.com122.x 15015736

[14] Do LG, Spencer AJ, Roberts-Thomson KF, Trinh HD, Nguyen TT (2011). Oral health status of Vietnamese children: findings from the National Oral Health Survey of Vietnam 1999. Asia Pacific Journal of Public Health.

[15] Pham TAV, Nguyen PA (2019). Factors related to dental caries in 10-year‐old Vietnamese schoolchildren. Int Dent J.

[16] Ngoc VTN, Chu D-T, Le D-H (2017). Prevalence of early childhood caries and its related risk factors in preschoolers: Result from a cross sectional study in Vietnam. Pediatr Dent J.

[17] Nguyen TT, Nguyen BBT, Nguyen MS, Olak J, Saag M (2016). Effect of School Oral Health Promotion Programme on dental health and health behaviour in Vietnamese schoolchildren. Pediatr Dent J.

[18] Lwanga SK, Lemeshow S (1991). Sample size determination in health studies: a practical manual.

[19] World Health Organization (2013). Oral health surveys: basic methods- 5th Edition.

[20] Kazeminia M, Abdi A, Shohaimi S, Jalali R, Vaisi-Raygani A, Salari N, et al. Dental caries in primary and permanent teeth in children’s worldwide, 1995 to 2019: a systematic review and meta-analysis. Head & Face Medicine. 2020;16(1). 10.1186/s13005-020-00237-z PMC7541284 33023617

[21] Al-Darwish M, El Ansari W, Bener A. Prevalence of dental caries among 12–14year old children in Qatar. 2014;26(3):115–25. 10.1016/j.sdentj.2014.03.006 PMC4095054 25057232

[22] Mahfouz M, Abu Esaid A (2014). Dental Caries Prevalence among 12–15 Year Old Palestinian Children. International Scholarly Research Notices.

[23] Ingle NA, Dubey HV, Kaur N, Gupta R (2014). Prevalence of dental caries among school children of Bharatpur city, India. J Int Soc Prev Community Dent.

[24] Subedi B, Shakya P, Kc U, Jnawali M, Paudyal BD, Acharya A (2011). Prevalence of dental caries in 5–6 years and 12–13 years age group of school children of Kathmandu valley. JNMA J Nepal Med Assoc.

[25] Laganà G, Fabi F, Abazi Y, Kerçi A, Jokici M, Nastasi EB (2012). Caries prevalence in a 7- to 15-year-old Albanian schoolchildren population. Ann Stomatol (Roma).

[26] Moreira PV, Rosenblatt A, Severo AM (2006). Prevalence of dental caries in obese and normal-weight Brazilian adolescents attending state and private schools. Community Dent Health.

[27] Watt R, Heilmann A, Listl S, Peres M (2016). London charter on oral health inequalities. J Dent Res.

[28] Petersen PE, Kwan S (2011). Equity, social determinants and public health programmes – the case of oral health. Commun Dent Oral Epidemiol.

[29] Schwendicke F, Dörfer CE, Schlattmann P, Page LF, Thomson WM, Paris S (2015). Socioeconomic Inequality and Caries:A Systematic Review and Meta-Analysis. J Dent Res.

[30] Suprabha BS, Rao A, Shenoy R, Khanal S (2013). Utility of knowledge, attitude, and practice survey, and prevalence of dental caries among 11-to 13-year-old children in an urban community in India. Glob Health Action.

[31] Al-Samadani K, Ahmad M, Bakeer H, Elanbya MO (2017). Oral health knowledge and practice among 9-12-year-old schoolchildren in the region of Madinah, Saudi Arabia, and its impact on the prevalence of dental caries. European Journal of General Dentistry.

[32] Prasai Dixit L, Shakya A, Shrestha M, Shrestha A (2013). Dental caries prevalence, oral health knowledge and practice among indigenous Chepang school children of Nepal. BMC Oral Health.

[33] Haque SE, Rahman M, Itsuko K, Mutahara M, Kayako S, Tsutsumi A (2016). Effect of a school-based oral health education in preventing untreated dental caries and increasing knowledge, attitude, and practices among adolescents in Bangladesh. BMC Oral Health.

[34] Kumar S, Tadakamadla J, Kroon J, Johnson NW (2016). Impact of parent-related factors on dental caries in the permanent dentition of 6–12-year-old children: A systematic review. J Dent.

[35] Antunes JLF, Narvai PC, Nugent ZJ (2004). Measuring inequalities in the distribution of dental caries. Commun Dent Oral Epidemiol.

[36] Marmot M, Bell R (2011). Social Determinants and Dental Health. Adv Dent Res.

[37] Reddy KS, Reddy S, Ravindhar P, Balaji K, Reddy H, Reddy A (2017). Prevalence of dental caries among 6–12 years school children of Mahbubnagar District, Telangana State, India: A cross-sectional study. Indian Journal of Dental Sciences.

[38] Youssefi MA, Afroughi S. Prevalence and Associated Factors of Dental Caries in Primary Schoolchildren: An Iranian Setting. International Journal of Dentistry. 2020;2020. 10.1155/2020/8731486 PMC7201520 32399035

[39] Kato H, Tanaka K, Shimizu K, Nagata C, Furukawa S, Arakawa M (2017). Parental occupations, educational levels, and income and prevalence of dental caries in 3-year-old Japanese children. Environ Health Prev Med.

